# Impact of life-threatening military incidents during deployments abroad on the relationships between military personnel and their families

**DOI:** 10.3389/fpsyt.2024.1419022

**Published:** 2024-07-18

**Authors:** Ulrich Wesemann, Katie Rowlands, Karl-Heinz Renner, Lucas Konhäuser, Kai Köhler, Hubertus Himmerich

**Affiliations:** ^1^ Department of Psychiatry, Psychotherapy and Psychotraumatology, Bundeswehr Hospital Berlin, Berlin, Germany; ^2^ Department of Psychological Medicine, Institute of Psychiatry, Psychology & Neuroscience (IoPPN), King’s College London, London, United Kingdom; ^3^ Institute of Psychology, Faculty of Human Sciences, Bundeswehr University Munich, Neubiberg, Germany

**Keywords:** family, military deployment, military personnel, intimate relationship, marital status, child, critical incident, Afghanistan

## Abstract

**Introduction:**

The influence of deployments on family relationships has hardly been investigated. Following a recently proposed new research strategy, military personnel with and without deployment-related life-threatening military incidents during deployment were compared. The hypothesis was that partner and family relationships of military personnel who experienced such an event would deteriorate more.

**Methods:**

This study included *N* = 255 military personnel who had a romantic partner (*n* = 78 of them had children) when deployed to Afghanistan. Of these, *n* = 68 military personnel experienced a deployment-related critical event during the deployment, *n* = 187 did not. Partnership quality was assessed using a semi-structured pre- and post-deployment interview.

**Results:**

The partner relationships of military personnel who experienced a deployment-related life-threatening military incident during deployment broke up significantly more often. The partner relationships of all military personnel deteriorated significantly, with greater deterioration after deployment in the group who faced such incidents. These results were independent of age, rank or number of previous deployments. In addition, there was a significant deterioration in the relationships between all military personnel and their children with greater deterioration after deployment in the group who faced such incidents.

**Conclusion:**

Life-threatening military incidents during a deployment abroad appear to have a considerable influence on the quality and stability of the partner and family relationships of military personnel. These findings can be used to inform the development of specific pre- and post-deployment measures and training.

## Introduction

Military deployments bear a high risk of being exposed to life-threatening incidents like combat exposure (1). These incidents can lead to or increase the likelihood of mental disorders (2–6), reduced quality of life including aggressive and violent behaviour (7), partnership problems (8, 9) or physical injuries (10–12). Thus, it can be assumed, that mental health impairment (13) as well as physical and mental disorders are part of the occupational risk of military personnel and other emergency responders (14, 15). In the past decades, many studies have been published on deployment related mental disorders among military personnel (16). Particular emphasis has been placed on post-traumatic stress disorder (PTSD), while other common mental disorders such as major depression or anxiety disorders have received more attention in recent years (17). There has also been increasing interest on resilience (18–21) and protective factors (15, 22) on the personalities of military personnel (23).

Incidence rates of mental disorders following deployments vary widely. This is attributed to different types of assessment, timing, military population, deployments, country/region, length of deployment, climatic conditions, attitude of the civilian population towards the soldiers, or changing danger situation (24). In Germany, the incidence for mental disorders after deployments range between 2.3% for PTSD, 2.4% for depression and 5.1% for anxiety disorders (24).

Mental disorders have an impact on intimate partner relationship in military families. In particular, alcohol problems, PTSD and depression are predictors for intimate partner violence (25). One systematic review focused on military personnel with mental disorders - regardless of deployment. The authors found negative influences on intimate relationships and psychological/psychosocial effects on the spouse (26).

In comparison to these occupational risks to mental health, relatively less research has focused on the impact of deployments abroad on family relationships. One of the first studies examined the association between combat-related deployments and child maltreatment. The risk of child maltreatment was around 1.4 times higher after deployment, while the risk of child neglect was around 1.6 times higher than before deployment (27). In a representative study from the UK, deployment had a negative impact on the intimate partner relationships of around 50% of military personnel and on their children. Additionally, those who were exposed to a life-threatening military incident were 1.4 times more likely to report negative effects on their relationships (28). Another study reported a 3.4% rate of intimate partner violence in military families after deployment (29). Taylor and colleagues found an increased risk of child maltreatment after deployment up to six months later. This was attributed to increased stress in the families of returning soldiers. The frequency of deployments played an important role. The risk of child maltreatment was increased after the first deployment, but not during or after the second (30).

Sullivan and colleagues found that the risk of severe family violence was about four times higher after a combat deployment (31). More than 12% reported physical violence toward family members and/or non-family members within three weeks of deployment. The risk doubled if they had an active combat role during deployment (32), while anger led to aggression and violence in the family (31, 33, 34). Another study found that combat deployments correlate with antisocial behaviour, which could explain this link (35). A meta-analysis summarising these results found an average monthly prevalence of “hitting someone” of 10% and an annual prevalence of 14% after deployment. The risk of aggressive or antisocial violent behaviour was about 3.2 times higher in military personnel with combat experience (7).

A recently published study (24) proposed comparing military personnel deployed to the same mission, with and without deployment-related life-threatening military incidents. In the group exposed to such incidents, the risk for developing PTSD, depression or anxiety disorders was six to seven times higher. Therefore, it is more likely that differences between these populations are due to the life-threatening incidents. The general stress factors of missions such as shifts in working hours, climate change, general risk situation, absence from home including separation from family, and others should also be taken into account.

Following this research strategy, we compared male combat military personnel with and without life-threatening military incidents during their deployment in Afghanistan. We hypothesized that military personnel who experienced life-threatening military incidents will report higher rates of breakups with their intimate partners, relationship problems with their intimate partners, and problems with their children. This is thought to be due to higher levels of traumatic stress, which often manifests as hyperarousal in the form of problematic anger (36). Traumatic stress activates the brain in a way that leads to persistent hyperarousal. The amygdala reacts to threats, while the prefrontal cortex, which regulates emotional responses, is underactive. This dysregulation leads to intense and uncontrolled emotions, including anger. Cognitive distortions such as catastrophising and personalisation reinforce the perception of threats and injustice and often lead to outbursts of anger. In addition, deficits in emotion regulation make it difficult to cope with these intense emotions, leading to problematic anger (37). This in turn has a significant impact on the partner and family relationships of military personnel after deployment (38, 39).

## Methods

All military personnel (*N* = 496) of a specific contingent of the German Armed Forces who were deployed in Afghanistan for six months in 2014 took part in the study. Of these, 353 military personnel had an intimate partner when data was first collected at the end of 2013. At the second measurement point, one year after the first assessment, *N* = 255 (72%) of the participants (*n* = 1 female; 0.4%) from the first measurement point could be examined again. Of these, a total of *n* = 68 (26.7%) had experienced a life-threatening military-specific event during the deployment (e.g. combat exposure, shelling from a distance, mines, engaged in close combat, aimed or shot at the enemy, or witnessed the injury or death of a comrade). The high dropout rate was due to changes in resource planning, job changes, further deployments and departures from active service (not due to physical or mental health problems).

The assessment of intimate partner and family relationships were carried out at both measurement points using a semi-structured interview (TIPSYFIT - Troop psychology interview for measuring psychological fitness). The life-threatening military incidents during deployment were recorded in the second assessment, one year after the first assessment. The interview was performed by troop psychologists who also completed one week of training in administering the interview. The interview was developed and evaluated for the German Armed Forces (Bundeswehr (40). Participation was mandatory for the entire project including the interview. Nevertheless, participants were informed that they had the right not to answer questions that were too intimate or stressful without giving reasons.

The partner relationship was assessed using the semi-structured interview (TIPSIFIT). First the question: “How would you describe the relationship between you and your partner currently?” was given. Relationship problems, time spent together, mutual support and sexual satisfaction were then assessed using open-ended questions. If answers were difficult to assess, examples were asked. The troop psychologist then used the information gathered to rate the quality of the relationship on one 5-point Likert scale ranging from 1 (very good) to 5 (very poor). Thus, the open questions were not documented or rated separately but were considered equally in the overall assessment.

The relationship between the military personnel and the children living in their household were assessed with the initial question, “How do you currently see the relationship between you and your children?”. The time spent together, conflicts and problem areas, parenting style and activities regularly carried out together were then assessed using open-ended questions. The overall assessment of the relationship was carried out by the psychologist in the same way as for the partnership.

Life-threatening military incidents during deployments were also assessed through the interview (TIPSIFIT) after deployment. The initial question was: “Have there been events … (during deployment) that you would spontaneously describe as stressful?” followed by open questions covering these events. Events were defined as type A criteria of posttraumatic stress disorder (F43.1) according to the International Classification of Diseases (ICD-10). The final assessment of life-threatening military incidents during deployment was dichotomous (event vs. no event).

The interview information was entered digitally and could not be changed independently after the next question. As the life-threatening military incident during deployment was only recorded at the end of the interview, it had no effect on the ratings of the quality of the partnership relationship or that of the children.

According to the Ministry of Defense’s specifications, the examination was mandatory for everyone. This related to the question of psychological fitness at the time. A separate ethics approval from the Bundeswehr University in Munich was therefore obtained for this secondary analysis (EK UniBw M 23-01).

Statistics: To examine group differences between participants with and without life-threatening military incidents during deployment, pre-deployment demographic data were compared using t-tests for independent samples. Group differences and odds ratios in terminated partnerships after deployment were calculated using chi-square tests. Due to the small sample size, no subgroup analyses were conducted for relationship status (married vs. not married). Changes in the relationships between the military personnel and their intimate partner, and with their children, were examined using separate time by group repeated measures analyses of variance (rmANOVAs). Although it can be assumed that a separation is preceded by a significant deterioration in the couple relationship, these people were not included in this calculation. This is due to the fact that this data is not available at the second measurement point and cannot be easily replaced. The reasons for the separation were not recorded. Finally, analyses of covariance (ANCOVAs) were conducted with the specific relationships (partner and children) as dependent variables, life-threatening military incidents as independent variables, and demographics as covariates. Due to the small number of female soldiers, gender-specific comparisons were not performed.

Statistical data calculations were conducted using IBM SPSS Statistics for Windows, Version 28.0 Armonk, NY, USA. The significance level was set at α ≤.05. According to the standards, an effect size of ƞ^2^ ≥.01 was considered small, of ƞ^2^ ≥.06 as medium and of ƞ^2^ ≥.14 as large (41).

## Results

To determine the socio-demographic differences before deployment in both groups, a t-test for independent samples was carried out. There were no differences in the two groups, meaning that the samples were comparable as shown in **Table 1**.

**Table 1:** t-test for group differences in demographic characteristics between soldiers with and without life-threatening military incidents during deployment.

	Deployment	*N*	Mean	SD	*T*-value	df	*p*-value
Age	With critical incident	67	26.7	4.75			
	Without	186	27.0	5.00	-0.41	251	.679
Rank	With critical incident	66	1.6	0.70			
	Without	186	1.5	0.66	-0.53	250	.605
N	With critical incident	68	0.7	1.02			
	Without	187	0.9	1.22	-0.98	253	.327

differences in N are due to missing data; SD, standard deviation; p-value, significance 2-tailed; N, number of previous deployments; YoS, years of service. Differences in df are due to missing data. df with decimals were corrected due to variance heterogeneity using Welch’s test.

A chi-square test was conducted to determine whether the group with life-threatening military incidents had a higher rate of relationship breakdowns after deployment. With χ^2^(1, *N*=255) = 4.9, *p* = .028, partner relationships in this group broke up significantly more often. The odds ratio (OR) = 2.0 [95% CI: 1.00-4.04] for partnership break-ups in the group with life-threatening military incidents was twice as high as in the group without life-threatening incidents.

To test for changes in relationships between the remaining couples, an rmANOVA was conducted. There was a main effect of Time, F(1, 216) = 4.1, p = .028, indicating that partnership worsened over time in the total sample. There was a trend (two tailed) towards a time by group interaction effect, with a worsening in the group with a life-threatening military incident: F(1, 216) = 3.4; p = .066, as shown in **Table 2**. Additionally, an ANCOVA was conducted to control for number of previous deployments, age, and rank. As shown in **Table 3**, only life-threatening military incidents remained significant, while all covariates did not.

**Table 2:** repeated measures ANOVA to test the influence of life-threatening military incidents during deployment on soldiers’ relationships with their partners.

Group	N	Mean t1	SD	Mean t2	SD	df	F	Sig.	ƞ^2^
With life-threatening military incidents	55	1.85	1.04	2.13	1.22	1			
Without life-threatening military incidents	163	1.41	0.72	1.44	0.67	1			
Main effect	218	1.52	0.83	1.61	0.89	216	4.88	.028	0.22
Interaction effect	218					216	3.40	.066	0.16

Df, degrees of freedom; F, F-value; Sig., significance; ƞ^2^, partial eta square; Main effect, total group (within subjects); Interaction effect, Time by group with vs. without life-threatening military incidents (between subjects).

**Table 3:** ANCOVA to test the influence of critical incidents during deployment on the partner relationship including number of previous deployments, age, and rank as covariates.

Predictors	df	F	Sig.	ƞ^2^
Number of previous Deployments	1	0.38	.537	.002
Age	1	0.01	.940	<.001
Rank	1	0.88	.349	.004
Critical Incident	1	22.72	<.001	.088

Df, degrees of freedom; F, F-value; Sig., significance; R^2^ = .10; ƞ^2^, partial eta square.

Despite the separations in the couples’ relationships, there were no separations in the relationship between the military personnel and their children (χ^2^ < 1; n.s.).

Finally, we assessed changes in the relationship of the military personnel with their children by comparing the groups with and without a life-threatening military incident using a rmANOVA. As shown in **Table 4**, there was a main and a time by group interaction effect indicating a general worsening in the relationship with a more severe change in the group with life-threatening military incidents. An ANCOVA that controlled for the number of previous deployments [*F*(1, 56) = 1.3, *p* = .255], age [*F*(1, 56) = 0.3, *p* = .588], and rank [*F*(1, 56) = 0.1, *p* = .779] showed only a significant contribution of life-threatening military incidents during deployment to the deterioration of the relationship with the children: *F*(1, 56) = 4.9, *p* = .031.

**Table 4:** repeated measures ANOVA to test the influence of life-threatening military incidents during deployment on soldiers’ relationships with their children.

Group	N	Mean t1	SD	Mean t2	SD	df	F	Sig.	ƞ^2^
With life-threatening military incidents	19	1.37	0.50	1.89	0.88	1			
Without life-threatening military incidents	59	1.41	0.62	1.42	0.62	1			
Main effect	78	1.40	0.59	1.54	0.72	76	8.12	.006	0.10
Interaction effect	78					76	7.14	.009	0.09

Df, degrees of freedom; F, F-value; Sig., significance; ƞ^2^, partial eta square; Main effect, total group (within subjects); Interaction effect, Time by group with vs. without life-threatening military incidents (between subjects).

## Discussion

*N* = 255 military personnel were examined before and after their deployment in Afghanistan. A comparison was made between *n* = 68 military personnel with and *n* = 187 personnel without a life-threatening military incident. It was found that the intimate partner relationships of military personnel who experienced such an incident broke-up two times more frequently than those of their comrades. This means that these occupational risks also have an impact on the private lives of military personnel and their partners. This information should be shared during partnership seminars that are routinely offered by the Bundeswehr after deployment. It could help partners of military personnel to avoid misunderstandings and develop more empathy for the changes after deployment, but without accepting aggressive or abusive behaviour.

Among personnel who remained in a relationship after deployment, we also found a significant deterioration. While this partnership deterioration was found across the entire group, we only found a trend (when comparing two-tailed) suggesting that partnerships were worse among personnel who had a life-threatening military incident. We think this result could be due to several factors. Perhaps our “risk sample” is too small to find such differences. Since this is a directional hypothesis, a one-sided *p*-value would have to be considered. Approximately twice as many of the partnerships in this group were terminated and their results were therefore dropped-out and no longer taken into account. Since separations often follow major partnership conflicts, this is at least very likely. The ANCOVA also provides evidence of this. If the number of previous deployments, age and rank were also taken into account, a significant deterioration in the couple relationship was only found in the case of life-threatening military incidents during the deployment. Similar results were found in a qualitative study carried out in Colombia. Here too, the partnerships deteriorated over time due to absence during military deployment. But this was all the more the case when there were critical military incidents or when they were frequently exposed to death or suffering (42). In addition, intimate partner violence appears to negatively affect autobiographical memory (43).

Our results indicated that the relationships between military personnel and their children deteriorated significantly after the Afghanistan deployment. This result was initially independent of events during the operation. In addition, there was also a group-by-time interaction effect. This means that the relationship in the group of personnel with a life-threatening military incident deteriorated more significantly than in the group without such events. This result is consistent with a previous study from the USA. Posttraumatic stress, problematic anger and depressive symptoms in military personnel after deployment had a negative impact on their relationships with their partners and children (38). This result is also supported by a Canadian study, which found a high prevalence of the coincidence of deployment-related traumatic events and child maltreatment (44).

This result should also be incorporated into the family seminars after the deployment. This information could prepare both the military personnel and their partners to such changes. This knowledge could lead to a quicker response in partners to changing towards inappropriate behaviour. In fact, this information could sensitize military personnel even earlier and make them aware of changes in their attitudes and feelings toward their children.

Limitations: This is a secondary analysis of a psychological fitness dataset. As the survey was mandatory for the original question, this could have had an influence on the response behaviour. Due to some missing data, the ANCOVA on changes in relationships between military personnel and their children includes fewer participants. This could have slightly distorted the result. As the overall sample is small, other covariates such as mental disorders before and after deployment, trauma history, relationship duration, age of children, relationship status (married, not married) or education were not examined. And finally, a distinction was only made between “objectively life-threatening military incidents” and “no objectively life-threatening incidents”. However, the subjective perception of a threat can lead to the same consequences. This question cannot be answered with the available data set. Although the life-threatening military incidents during deployment were only assessed at the end of the interview, it cannot be ruled out that some of this information was already known beforehand. This could then have led to a falsification of the assessments. We suspect problematic anger as the main cause of the deterioration in relationships. However, since this was not explicitly recorded in this study, no further statement can be made.

## Conclusion

It is known that deployments convey a high occupational risk of developing mental disorders. It is also known that mental disorders are often accompanied by problems in relationships and families. The results of this study indicate that life-threatening military incidents during the deployment may increase the risk for family problems and relationship breakdowns. We suspect that problematic anger, hostility and paranoid thoughts are the main causes of the deterioration in relationships. These changes have been frequently observed in firearm-carrying emergency personnel after critical incidents (38, 45, 46). However, as this was not recorded, no definitive statement can be made.

It is therefore important for future missions to address these family problems in pre- and post-mission preparations (18, 47). In order to implement this properly, it seems necessary to offer special training and information events (48). These should not only be aimed at the military personnel, but also at their families. The support that the psychological service and the social service already offer should be significantly expanded accordingly. In addition to these existing programs, special programs should be established to address these issues. However, our current study has only identified the increased risk of family problems after a military incident during deployment, and therefore a rationale for the development of interventions that target those problems. It does not provide evidence for the effectiveness of such interventions in this population. Since these relationship and child problems are related to the life-threatening military incidents during the deployment, the military and politicians who make decisions on military engagements in conflict zones bear responsibility for military families. This also brings the need for better networking of military-civilian health care to the fore (49, 50).

## Data availability statement

Given the sensitivity of the mandatory study design, the data were not made publicly available. Data requests can be directed to the corresponding author. They are then checked on a case-by-case basis and require the approval of the Federal Ministry of Defence. Requests to access these datasets should be directed to uw@ptzbw.org.

## Ethics statement

The studies involving humans were approved by Bundeswehr University Munich (No: EK UniBw M 23-01). The studies were conducted in accordance with the local legislation and institutional requirements. Written informed consent for participation was not required from the participants or the participants’ legal guardians/next of kin because this study was a secondary analysis of a dataset.

## Author contributions

UW: Conceptualization, Data curation, Formal analysis, Funding acquisition, Investigation, Methodology, Project administration, Writing – original draft. KR: Resources, Supervision, Validation, Writing – review & editing. KR: Resources, Supervision, Validation, Writing – review & editing. LK: Validation, Writing – review & editing. KK: Resources, Supervision, Validation, Writing – review & editing. HH: Methodology, Resources, Supervision, Validation, Writing – review & editing.
